# Liver metabolic changes induced by conjugated linoleic acid in calorie-restricted rats

**DOI:** 10.1590/2359-3997000000186

**Published:** 2016-08-23

**Authors:** Camila de Moraes, Camila Andrea de Oliveira, Maria Esméria Corezola do Amaral, Gabriela Arcurio Landini, Rosana Catisti

**Affiliations:** 1 Centro Universitário Hermínio Ometto, Uniararas Araras SP Brazil Programa de Pós-Graduação de Ciências Biomédicas, Centro Universitário Hermínio Ometto, Uniararas, Araras, SP, Brazil

**Keywords:** Trans10cis12-conjugated linoleic acid, mitochondria, liver, restriction, caloric, metabolism, liver, metabolism, connexins

## Abstract

**Objective:**

Complexes like conjugated linoleic acid (CLA) reduce the percentage of body fat by increasing energy expenditure, fat oxidation, or both. The aim of this study was to verify if CLA is able to mimic caloric restriction (CR), and determine the effects of CLA on liver metabolic profile of young adult male Wistar rats.

**Materials and methods:**

We divided 36 animals into the following groups: 1) Control; 2) CLA (1% of daily food intake, 21 days, orogastric intubation); 3) Restr (fed 60% of the diet offered to controls); and 4) CLA Restr. Liver tissues were processed for biochemical and molecular or mitochondrial isolation (differential centrifugation) and blood samples were collected for biochemical analyses.

**Results:**

Treatment of the animals for 21 days with 1% CLA alone or combined with CR increased liver weight and respiration rates of liver mitochondria suggesting significant mitochondrial uncoupling. We observed a decrease in adipose tissue leading to insulin resistance, hyperinsulinemia, and hepatic steatosis due to increased liver cholesterol and triacylglycerol levels, but no significant effects on body mass. The expression of hepatic cellular connexins (43 and 26) was significantly higher in the CLA group compared with the Control or Restr groups.

**Conclusion:**

CLA does not seem to be a safe compound to induce mass loss because it upregulates the mRNA expression of connexins and induces hepatic mitochondrial changes and lipids disorders.

## INTRODUCTION

Studies on the reduction of dietary caloric intake without lack of essential nutrients, called caloric restriction (CR), have shown that this intervention can modulate biochemical pathways and prevent molecular diseases ( 1 ). In mammals, CR stimulates respiration rates ( 1 ), increases biogenesis and mitochondrial density in tissues ( 2 ), and decreases the coupling between oxygen uptake and oxidative phosphorylation ( 3 ). CR promotes mitochondrial biogenesis through a pathway signaled by lower insulin levels, increased nitric oxide production, and activation of the transcriptional coactivator PGC-1α. In addition, CR is associated with lower mitochondrial reactive oxygen species (ROS) generation, possibly due to enhanced uncoupling promoted by this dietary intervention ( 4 ). The broad spectrum of actions and marked metabolic and hormonal changes induced by CR have encouraged the identification of natural and synthetic compounds that mimic the effects of CR. The discovery of compounds that attenuate diseases of old age could have a profound impact on public health, reducing the incidence of diseases, increasing quality of life, and extending longevity. One of these compounds that has been extensively studied is conjugated linoleic acid (CLA, dienoic isomers of linoleic acid).

Some studies ( 5 ) but not all ( 6 ) have shown that CLA may reduce adiposity and suppress weight gain in humans. The health benefits attributed to CLA include anticarcinogenic activity ( 7 ), antiatherosclerotic effects ( 8 ), modification of the composition and metabolism of adipose tissue ( 9 ), immune response modulation ( 10 ), and increased glucose and insulin tolerance ( 11 ). Some investigators have proposed that CLA reduces body fat percentage by increasing energy expenditure in AKR/J mice without increasing the uncoupling protein gene expression ( 12 ), fat oxidation, or both ( 13 ). However, the cause of increased fat oxidation remains unknown, and it is still unclear whether it affects ingested or endogenous fat. Some studies suggest that mitochondrial oxidative capacity is altered in liver disease ( 14 , 15 ). Substantial published data have reported the effect in liver mitochondria of CLA not associated with CR ( 16 - 18 ). In skeletal muscle cells, CLA increases mitochondrial biosynthesis ( 19 ), a similar effect promoted by CR.

Previous studies have determined the occurrence of upregulation and redistribution of beta-catenin and E-cadherin in MCF-7 breast cancer cells ( 20 ) with CLA treatment. The coordinated integration of extracellular, intracellular, and intercellular mechanisms promotes the maintenance of homeostasis in higher organisms. The establishment of communicative networks between the different liver cell types is, therefore, indispensable. Hepatocytes, the most prominent liver cell population, communicate directly with each other through gap junctions (connexins) and adhesion molecules (cadherins) ( 21 ). An issue that has been investigated is whether treatment with CLA and/or CR could interfere with the cellular integrity of the liver tissue through modulation of connexins and cadherins that may impair important cellular functions, such as migration, adhesion, and cell cycle.

The objective of the present study was to investigate the role of CLA alone or combined with CR on liver metabolism. We first analyzed the mitochondrial oxidative stress to verify if CLA, as a mitochondrial uncoupler, would mimic CR. We specifically studied energy parameters measured by mitochondrial swelling and oxygen uptake in hepatic mitochondria isolated from control rats and rats submitted to 40% CR supplemented or not with CLA. We also measured serum glucose, insulin, total protein and lipid levels, muscle and liver glycogen, and weight changes in hepatic and periepididymal adipose tissues, and investigated the integrity and homeostasis maintenance in liver tissue by analyzing the expression of connexins and cadherins transcripts.

## MATERIALS AND METHODS

### Animal care

All experiments were conducted in strict agreement with the Guide for the Care and Use of Laboratory Animals and were approved by the local Animal Care and Use Committee (Permit No. 313/2009). Eight-week-old male Wistar rats were maintained in individual metabolic cages on a 12-h light/12-h dark cycle at a controlled temperature (21 ± 1^o^C), with free access to food and water. After an adaptation period of 24 h, we measured the food and water intake of the animals for an additional 24 h. No differences in food and water intake between the treated and untreated groups were observed at any time. Thirty-six animals were randomly divided into four groups of nine animals each: 1) Control group; animals fed a standard diet (Nuvilab CR-1, Nuvital, Colombo, PR, Brazil) and water by orogastric intubation; 2) CLA group; animals fed a standard diet and treated with CLA; 3) Restr group; animals fed 60% of the diet consumed by control animals and water by orogastric intubation; 4) CLA Restr group; animals fed 60% of the diet consumed by control animals and treated with CLA. The groups received the commercial CLA mixture 75% AdvantEdge^®^ CLA (EAS^TM^ Golden, CO, USA) or water at a concentration corresponding to 1% of daily food intake. The fatty acid composition of the commercial mixture 75% AdvantEdge^®^ CLA, expressed in g/100 g of fatty acids, is detailed in ( 22 ). Briefly, fatty acid composition of CLA (g/100 g of fatty acids): 0.75 of C18:2 *cis* -9, *cis* -12; 40.12 of C18:2 *cis* -9, *trans* -11 CLA; 39.15 C18:2 *trans* -10, *cis* -12 CLA. The animals were supplemented daily by orogastric intubation using disposable 1-mL syringes and gavage needles. The amount of supplement administered was calculated every 2 days based on the mean daily food intake in each group. The density of each supplement was taken into account for the calculation of the quantity in milliliters (approximately 0.1 to 0.6 mL). Body weight and food intake were recorded weekly.

### Experimental procedures

Rectal temperature was measured with a digital thermometer (BD Basic, Becton Dickinson, São Paulo, Brazil). Temperatures were recorded between 2 and 3 pm once a week. In addition, the animals were weighed individually once a week. After 21 days of treatment, the four groups of six animals were sacrificed for mitochondrial isolation after a 12-h fast by cervical dislocation performed by a technician experienced in the procedure. For biochemical analysis (four groups of three animals), blood samples were obtained after anesthesia by heart puncture and sera were stored at -20°C. Liver tissues were collected and weighed, and fragments were processed for histological and biochemical analysis. Serum glucose, total protein, cholesterol, and triacylglycerol levels were measured using commercial kits according to the manufacturer’s instructions (Laborlab, São Paulo, Brazil). Insulin was measured by ELISA (Linco Research, St. Charles, MO, USA). Hepatic and muscle glycogen were determined as described elsewhere ( 23 ). Liver total lipids were extracted according to the method of Folch and cols. ( 24 ).

### Histology

Livers were removed and their fragments were immersed in a fixative solution containing 10% formaldehyde in Millonig buffer, pH 7.4, for 24 h at room temperature. Next, the specimens were washed in buffer and submitted to standard procedures for embedding in Paraplast^®^ (Merck, Darmstadt, Germany). Longitudinal 6-µm thick sections were stained with hematoxylin-eosin. The slides were analyzed and documented under a Leica DM2000 photomicroscope at the Laboratory of Micromorphology/Uniararas.

### Intraperitoneal glucose tolerance test

After 21 days of treatment, the four groups of three animals were fasted overnight (16-18 h), weighed, and injected intraperitoneally with d-glucose (Sigma-Aldrich, St. Louis, MO, USA) at a dose of 2 g/kg of body weight. Blood samples were collected by cutting the tip of the tail at 0, 30, 60, and 120 min after glucose injection. Serum glucose was determined using test tapes (MediSense Optium Xceed, Abbott Laboratories, CA, USA). The glucose response was calculated by estimating the total area under the curve using the trapezoidal method ( 25 ). Liver mitochondria were not isolated from these animals.

### Isolation of rat liver mitochondria

The livers were weighed immediately after sacrifice. Rat liver mitochondria (RLM) were isolated from the livers of overnight-fasted adult Wistar rats by conventional differential centrifugation according to ( 26 ). RLM samples were homogenized for the determination of protein content. All experiments using isolated mitochondria were conducted within 1 h of isolation.

### Standard incubation procedure

The RLM experiments were carried out at 28^o^C in a reaction medium containing 125 mM sucrose, 65 mM KCl, 10 mM HEPES buffer, pH 7.2, 1 mM inorganic phosphate, 2 mM sodium succinate, 5 µM rotenone, and 10 µM CaCl_2_. Rotenone, sodium succinate, and HEPES were purchased from Sigma-Aldrich (St. Louis, MO, USA).

### Determination of mitochondrial swelling and oxygen uptake

Mitochondrial swelling and oxygen uptake were performed according to established protocol ( 27 ). The variation in absorbance at 540 nm was measured with a Genesys 10UV spectrophotometer (Thermo Electron Corporation, Madison, WI, USA), and oxygen uptake was monitored with a Clark-type electrode (Oxytherm System, Hansatech Instruments, Norfolk, UK). Briefly, RLM (0.5 mg/mL) were incubated in standard reaction medium. Inorganic phosphate (2 mM) was added after 1 min of mitochondrial preincubation and absorbance was recorded over a period of 10 min. The mitochondrial swelling and oxygen uptake experiments were performed simultaneously using the same preparation of isolated RLM under the same experimental conditions. Mitochondrial respiration (oxygen uptake) was recorded over a period of 10 min, assuming a solubility of 210 µmol/mL at 28^o^C.

### RNA isolation and semiquantitative reverse transcriptase-PCR (RT-PCR)

Total RNA was isolated from approximately 100 mg of rat liver with the TRIzol^®^ reagent (Invitrogen, CA, USA) and digested with DNAse I, Amplification Grade (Invitrogen) according to the manufacturer’s instructions. RNA concentration was determined by measuring UV absorbance at 260 nm using a spectrophotometer, and integrity was confirmed by formaldehyde gel electrophoresis. The total RNA samples were stored at -80°C until further use for analysis. cDNA was synthesized from 2 µg of RNA in the presence of dithiothreitol, dNTP, random primers, RNAseOUT, and SuperScript™ II Reverse Transcriptase (Invitrogen) in a final volume of 20 µL. The mRNA levels of the E-cad, N-cad, Cx26, Cx32, and Cx43 genes were investigated by semiquantitative RT-PCR. Primer sequences used in the PCR reactions were chosen based on the sequences available in GenBank. E-cad was amplified using gene-specific forward (5’-GCAGTTCTGCCAGAGAAACC-3’) and reverse (5’-AATCCTGCTTCCAGGGAGAT-3’) primers with an expected amplicon of 315 bp (Tm 55°C). The primers for N-cad (forward primer 5’-TGTTGCTGCAGAAAACCAAG-3’ and reverse primer 5’-GGCGACTCTCTGTCCAGAAC-3’) amplified a predicted amplicon of 309 bp (Tm 53°C) while the primers used for Cx26 (forward primer 5’-GGTGTGGGGAGATGAGCAAG-3’ and 5’-GACTTCCCTGAGCAATACCT-3’) had an expected amplicon of 540 bp (Tm 62°C). Cx32 was amplified using gene-specific primers (Tm 57°C; forward primer 5’-AATGAGGCAGGATGAACTGG-3’ and reverse primer 5’-CCTCAAGCCGTAGCATTTTC-3’) and resulted in the amplification of the predicted 339 bp product. Cx43 was amplified using gene-specific forward (5’-GATTGAAGAGCACGGCAAGG-3’) and reverse (5’-GTGTAGACCGCGCTCAAG-3’) primers with an expected amplicon of 144 bp (Tm 58°C). ACTB (β-actin) was used as a housekeeping gene (Tm 57°C; forward primer 5’-AGAGGGAAATCGTGCGTGACA-3’ and reverse primer 5’-CGATAGTGATGACCTGACCGTCA-3’) yielding an amplification product of 178 bp that was used to normalize connexins and cadherins mRNA levels.

The amplified products were separated on 1.5% agarose gel stained with ethidium bromide, visualized, and photographed by the gel documentation system Syngene G: Box^®^. Signal intensities of the bands were measured densitometrically using the Scion Image software. Each value was determined as the mean of three densitometric readings. The results are expressed as average ratios of the relative optical densities of E-cad, N-cad, Cx26, Cx32, and Cx43 PCR products in relation to β-actin.

### Data analysis

**Figure 1:**
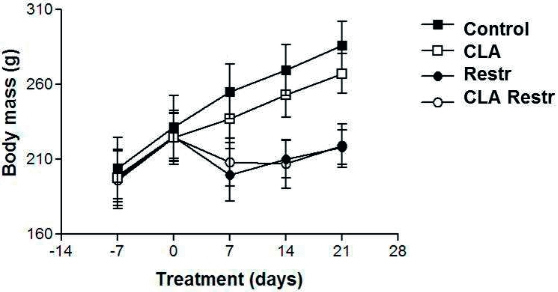
CLA treatment had no effect on body mass. Body mass was recorded weekly in Control (full square symbols), Restricted (Restr, full circle symbols), CLA-treated (CLA, empty square symbols), and restricted CLA-treated (CLA Restr, empty circle symbols) animals. Time 0 represents the start of treatment (* p < 0.05 versus Control group, n = 9).

**Figure 2:**
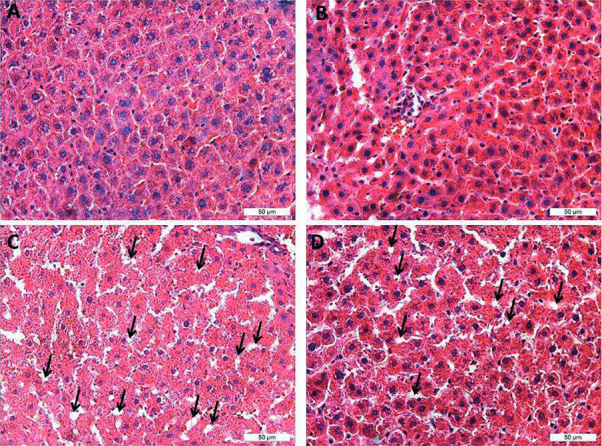
Photomicrographs of sagittal liver sections stained with hematoxylin-eosin. Tissue sections of 4.0 mm are shown in panels A (Control), B (Restr), C (CLA), and D (CLA Restr). Magnification X50. Arrows indicate fat vesicles.

**Figure 3:**
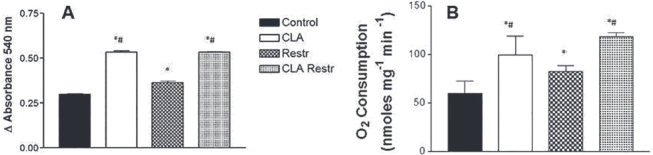
CLA increased mitochondrial swelling and oxygen consumption. Rat liver mitochondria (RLM; 0.5 mg/mL) were added to a standard reaction medium containing 125 mM sucrose, 65 mM KCl, 10 mM HEPES buffer, pH 7.2, 10 µM CaCl2, 1 mM iP, 2 mM succinate, 5 µM rotenone, at 28oC, and the (A) variation in 540-nm absorbance (B) and oxygen uptake were measured over a period of 10 min, as described in the Materials and Methods section. Inorganic phosphate (1 mM) was added after 1 min of mitochondrial preincubation (p < 0.05; * versus Control, # versus Restr, n = 6).

Figures 1 - 3 report the mean ± standard deviation (SD) of measurements from six different animals. Data were compared by one-way ANOVA followed by Tukey’s *post hoc* test performed using GraphPad Prism software (GraphPad Software, Inc., La Jolla, CA, USA) adopting a level of significance of 5% ( *p* < 0.05).

## RESULTS

### Characteristics of the animals

The body weight gain of the animals was analyzed weekly (0, 7, 14, and 21 days) during the treatment period ( Figure 1 ). The results of the Restr and CLA Restr groups showed a significant reduction in body mass from day 7 to 21 of treatment compared with the CLA and Control groups. The ratio of liver weight and body weight (LW/BW) ( Table 1A ) showed that CLA treatment significantly increased the liver mass. The ratio of gonadal adipose tissue weight and body weight (AW/BW) showed a significant reduction promoted by CLA treatment. There were no differences in total protein, liver glycogen, or area under the glucose curve in the intraperitoneal glucose tolerance test between groups. Glucose, insulin, liver cholesterol, and triacylglycerol levels were lower in rats exposed to CR when compared with control animals. Treatment for 21 days resulted in an increase in insulin and total liver cholesterol, and a decrease in muscle glycogen and AW/BW index in the CLA and CLA Restr groups compared with the Control group. Serum triacylglycerol and cholesterol levels did not differ between the CLA and CLA Restr groups.

**Table 1:** Serum levels of fasting glucose, insulin, cholesterol, triacylglycerol, total protein; liver cholesterol, triacyglycerol, glycogen, and glycogen muscle and adipose tissue of control and of rats maintained on CR (Restr) or CLA treated (CLA and CLA Restr) for 21 days

Parameter	Control	Restr	CLA	CLA Restr
Body weight BW (g)	277.4 ± 42.06	215.40* ± 35.53	257.00 ± 53.28	215.2* ± 44.53
Liver weight LW (g)	10.12 ± 0.3481	6.565* ± 0.3951	11.41*^#^ ± 1.285	7.685* ± 0.09152
LW/BW (%)	3.65 ± 0.02	3.05* ± 0.03	4.44* ± 0.03	3.57^#^ ± 0.02
Gonadal adipose tissue weight AW (g)	1.1 ± 0.11	0.77* ± 0.11	0.85 ± 0.26	0.62* ± 0.15
AW/BW (%)	0.39 ± 0.02	0.35 ± 0.03	0.33* ± 0.01	0.28*^#^ ± 0.02
Glucose (mmol/L)	5.39 ± 0.5	3.84* ± 0.23	5.11^#^ ± 0.21	4.12 ± 0.22
Insulin (U/mL)	0.39 ± 0.12	0.21* ± 0.05	1.4* ± 0.22	1.7* ± 0.5
Cholesterol (mg/dL)	168.00 ± 9.3	129.01* ± 21.00	171.00^#^ ± 19.00	179.00^#^ ± 20.00
Triacylglycerol (mg/dL)	91.00 ± 18.00	70.00* ± 6.00	125.00^#^ ± 15.01	121.01^#^ ± 7.00
Total protein (g/dL)	8.66 ± 0.66	9.64 ± 0.56	9.02 ± 0.62	8.91 ± 0.34
Liver cholesterol (mg/g tissue)	16.9 ± 2.5	10.8* ± 2	22.7*^#^ ± 3.9	31.4*^#^ ± 2.6
Liver triacylglycerol (mg/g tissue)	20.01 ± 6.5	9.35* ± 3.2	27.5*^#^ ± 3.5	23.00^#^ ± 4.3
Muscle glycogen (g/100 g tissue)	0.44 ± 0.02	0.45 ± 0.03	0.35*^#^ ± 0.17	0.32*^#^ ± 0.08
Liver glycogen (g/100 g tissue)	3.34 ± 0.33	2.45 ± 0.24	3.39 ± 0.7	2.09 ± 0.9
Area under glucose curve	15830 ± 127	16100 ± 179	15480 ± 132	15840 ± 156

p < 0.05 * vs Control; # vs Restr (Mean ± SD; n = 6-9).

### Liver histology

Photomicrographs of livers of Control (A), Restr (B), CLA (C), and CLA Restr (D) rats are shown in Figure 2 . The normal structure of the hepatocytes ducts, with visible improvement in tissue organization, can be seen in Figure 2B when compared with 2A. Some pyknotic nuclei and loss of cell and tissue definition, suggestive of mild steatosis (black arrows), were observed in the liver of control rats treated with CLA ( Figure 2C ). Discrete cell and structural organization were noted in the liver of CLA Restr ( Figure 2D compared with 2C).

### CLA-stimulated mitochondrial swelling and oxygen uptake

As seen in Figure 3A , Ca^2+^-induced mitochondrial swelling, measured by variation of absorbance in arbitrary units, was stimulated in RLM isolated from CLA (0.5340#* ± 0.012 ), Restr (0.3754* ± 0.004), and CLA Restr (0.5330#* ± 0.003) animals compared with those in the Control group (0.2970 ± 0.004) (* versus Control; # versus Restr, p < 0.05, n = 6). Under the same conditions, CR increased respiration in isolated mitochondrial preparations ( Figure 3B ). The results of liver oxygen uptake were 72.96 ± 3.154 nmol O_2_ mg^-1^ min^-1^ in the Control group, 109.5* ± 9.509 nmol O_2_ mg^-1^ min^-1^ in CLA-treated animals, 87.02* ± 2.171 nmol O_2_ mg^-1^ min^-1^ in the Restr group, and 137.42*^#^ ± 2.602 nmol O_2_ mg^-1^ min^-1^ in the CLA Restr group.

### Modulation of connexins and cadherins in the liver

**Figure 4:**
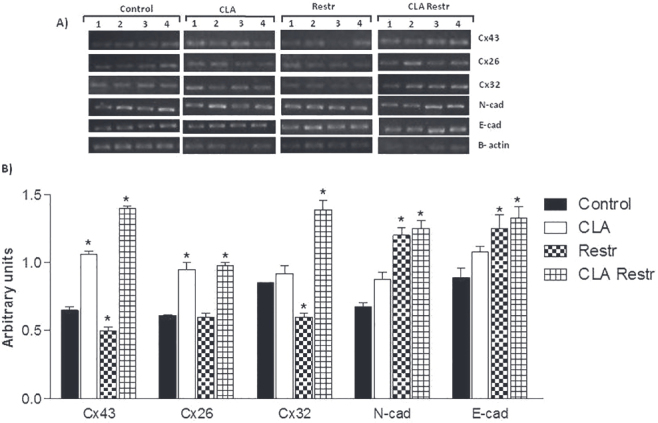
Liver mRNA levels of connexins (Cx) and cadherins (cad). (A) Representative semiquantitative RT-PCR of mRNA expression of Cx43, Cx32, Cx26, N-cad, and E-cad. (B) Bars represent densitometric analyses of connexins and cadherins mRNA expression in control and treated animals. Changes in mRNA are expressed as normalized densitometric units relative to *β* -actin mRNA. Values are represented as mean ± standard error of the mean (SEM). * P < 0.05 indicates statistical significance.

Compared with the Control group, the CLA Restr group showed significant increases in mRNA expression levels of connexin 43 (0.65 ± 0.026 versus 1.4 ± 0.019, respectively), connexin 26 (0.61 ± 0.07 versus 0.98 ± 0.022, respectively), connexin 32 (0.85 ± 0.003 versus 1.39 ± 0.07, respectively), N-cad (0.67 ± 0.03 versus 1.25 ± 0.06, respectively), and E-cad (0.89 ± 0.07 versus 1.33 ± 0.08, respectively) ( Figure 4 ). Similarly, hepatic mRNA expressions of connexins 43 (1.06 ± 0.025) and 26 (0.95 ± 0.05) were significantly increased in the CLA group compared with the Control group (0.65 ± 0.026 and 0.61 ± 0.07, respectively). In contrast, CR caused a significant reduction in connexins 43 (0.49 ± 0.030) and 32 (0.6 ± 0.03) mRNA and an increase in N-cad (1.2 ± 0.06) and E-cad (1.25 ± 0.1) levels compared with the Control group.

## DISCUSSION

Body weight gain analysis of the animals in our study showed that those in the Restr groups lost weight during the treatment period. These results validate the CR model: Restr animals presenting lower weight gain compared with Control ones, but with upward curves indicating animal growth. Body weight gain was similar in Control and CLA animals during the 3-week monitoring period. Weight gain was lower in Restr and CLA Restr animals. These results show that CLA had no effect on the body weight of the animals under the present experimental conditions. Biochemical analysis findings agree with the literature and suggest that CLA-treated animals had hyperinsulinemia in the presence of normoglycemia and presented no changes in plasma cholesterol or triacylglycerol, features of insulin resistance ( 28 ). A small reduction in liver glycogen and a significant reduction in muscle glycogen were observed in CLA-treated animals. This glycogen reduction is usually seen in hyperinsulinemic animals ( 29 ).

The significant increase in the LW/BW index found in rats treated with CLA may indicate physiological changes consistent with hepatic steatosis. These data were demonstrated by photomicrographs of sagittal sections of liver stained with hematoxylin-eosin ( Figure 2 ). In fact, these were confirmed by the results in Sprague-Dawley rats suggesting that CLA accelerates the decomposition of storage lipids, resulting in lipid peroxidation and morphological change in the liver ( 30 ). The presence of fat and liver cell alterations suggests hepatic steatosis and intoxication by CLA ( 30 , 31 ). In contrast, liver cells were intact in Restr animals, and CLA Restr animals showed less cell damage than CLA ones, suggesting a protective effect of CR. Our results show that the LW/BW ratio decreased in Restr, increased in CLA, and remained the same in CLA Restr animals compared with those in the Control group. Conversely, we observed a decrease in the AW/BW ratio in the CLA group, an effect enhanced by the CR. The body weight reduction in the Restr group can be explained by the reduction in liver weight, and in the CLA Restr group, by the reduction in adipose tissue weight. Taken together, these data may explain the liver damage caused by treatment with CLA.

The study of inner mitochondrial membrane permeability (MMP) induced by Ca^2+^ can be associated with a nonspecific increase in membrane permeability that stimulates respiratory rates and decreases the coupling between oxygen consumption and oxidative phosphorylation ( 32 ). The results suggest that the RLM CLA, Restr, and CLA Restr groups were more susceptible to the same MMP transition conditions than animals in the Control group. With the same stimulus to induce oxidative damage (10 µM CaCl_2_ and 2 mM inorganic phosphate), RLM from CLA, Restr, and CLA Restr animals exhibited a higher respiration rate. The uncoupling effect of CLA treatment on mitochondrial respiration was confirmed by the results of liver oxygen uptake ( Figure 2 ). As expected, CR increased respiration in isolated mitochondrial preparations, promoting mild mitochondrial uncoupling and proportional swelling, an effect that was enhanced in the CLA-treated groups. Kowaltowski and cols. have elegantly demonstrated that mild mitochondrial uncoupling is a highly effective *in vivo* antioxidant strategy, and that murine lifespan can be extended by low doses of the mitochondrial uncoupler 2,4-dinitrophenol in a manner accompanied by weight loss and lower serum levels of glucose, insulin, and triacylglycerol, as well as a pronounced decrease in biomarkers of oxidative damage and tissue ROS release ( 33 ). In the present study, the CLA-induced mitochondrial uncoupling activity was probably promoted by hepatic steatosis due to increased liver lipids content and had no effect on weight loss.

The increased hepatic cholesterol observed in CLA and CLA Rest animals may also explain the increases in connexin 43 and 26 mRNA expression in these groups. Also, the reduction in connexin 43 and 32 may be related to a decrease in cholesterol in the Restr group. Specific phospholipids are associated with different connexin isoforms, which suggests connexin-specific regulatory and/or structural interactions with lipid membranes and a potential role of membrane cholesterol in gap junction assembly and function ( 34 ). Cadherins are a superfamily of calcium-dependent adhesion molecules that play multiple roles in morphogenesis. A reduction in E-cadherin, in particular, is associated with invasion, increased cell proliferation, and metastasis ( 35 ). Expression levels of N-cadherin and E-cad mRNA were increased in Restr and CLA Restr animals compared with Control ones. This fact may suggest an antiproliferative effect of hepatocytes mediated by E-cadherin induced by CR, with no effect of CLA treatment. Therefore, our data are consistent with the literature and suggest that the increase in cadherins in animals undergoing CR contributes to decrease hepatic cell damage. Taken together, the present results suggest that CLA supplementation stimulates the accumulation of fat in the liver and increases insulin levels. CLA does not seem to be a safe mass loss compound because it induces hepatic mitochondrial and plasma lipid disorders, and upregulates the mRNA expression of connexins. The understanding of the biochemical mechanisms underlying the effect of CLA is important for the correct formulation of dietary interventions and adequate administration of food supplements.
